# Oxidative Stress in Ageing and Chronic Degenerative Pathologies: Molecular Mechanisms Involved in Counteracting Oxidative Stress and Chronic Inflammation

**DOI:** 10.3390/ijms23137273

**Published:** 2022-06-30

**Authors:** Thobekile S. Leyane, Sandy W. Jere, Nicolette N. Houreld

**Affiliations:** Laser Research Centre, Faculty of Health Sciences, University of Johannesburg, P.O. Box 17011, Doornfontein 2028, South Africa; sadie.leyane@gmail.com (T.S.L.); sandywjere@gmail.com (S.W.J.)

**Keywords:** oxidative stress, reactive oxygen species (ROS), inflammation, Alzheimer’s disease, Parkinson’s disease, diabetes, chronic kidney disease, antioxidants, stem cell therapy

## Abstract

Ageing and chronic degenerative pathologies demonstrate the shared characteristics of high bioavailability of reactive oxygen species (ROS) and oxidative stress, chronic/persistent inflammation, glycation, and mitochondrial abnormalities. Excessive ROS production results in nucleic acid and protein destruction, thereby altering the cellular structure and functional outcome. To stabilise increased ROS production and modulate oxidative stress, the human body produces antioxidants, “free radical scavengers”, that inhibit or delay cell damage. Reinforcing the antioxidant defence system and/or counteracting the deleterious repercussions of immoderate reactive oxygen and nitrogen species (RONS) is critical and may curb the progression of ageing and chronic degenerative syndromes. Various therapeutic methods for ROS and oxidative stress reduction have been developed. However, scientific investigations are required to assess their efficacy. In this review, we summarise the interconnected mechanism of oxidative stress and chronic inflammation that contributes to ageing and chronic degenerative pathologies, including neurodegenerative diseases, such as Alzheimer’s disease (AD) and Parkinson’s disease (PD), cardiovascular diseases CVD, diabetes mellitus (DM), and chronic kidney disease (CKD). We also highlight potential counteractive measures to combat ageing and chronic degenerative diseases.

## 1. Introduction

Ageing is a progressive and multifaceted physiological process characterised by the accretion of various degenerations in cellular and molecular structures, leading to deteriorated biological events and a gradual decline in the adaptability and resistance to metabolic stress. With ageing, there is a gradual decline in the body’s physical and mental capability to operate optimally [1]. The survival aptitude of a population is characterised by the decline in fertility and the survivorship curve. As this demographical drift continues, the constitution of the global population is projected to undergo a proportionate deviation such that the older generation outnumbers the younger generation. According to the World Health Organisation (WHO), in 2015, the average life expectancy increased to an average of 71.4 years, and from 2015 to 2050, the global population aged 60 years and above is expected to grow from 12% to 22% [2]. Although the populations’ ageing illuminates a positive civilization development, the continuation of this trend attributes to the functional deficit and increased susceptibility to disability and chronic diseases such as, but not limited to, diabetes, vascular ageing disorders, Alzheimer’s disease (AD), cardiovascular diseases (CVD), cancer, and muscle dysfunction [3,4,5].

**Figure 1:**
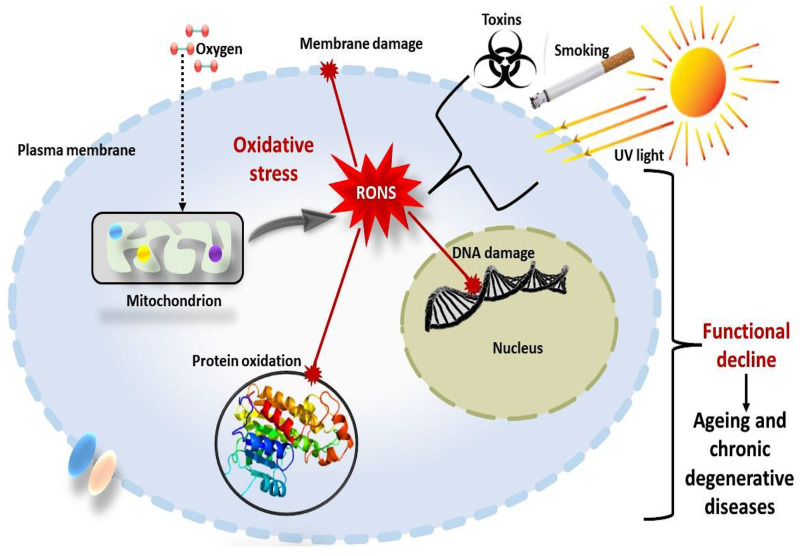
Schematic representation of oxidative stress, a phenomenon elevated with ageing and degenerative diseases. It involves the accumulation of reactive oxygen and nitrogen species (RONS) in cells and tissues, harmfully modifying deoxyribonucleic acid (DNA), proteins and lipids and triggering ageing and chronic degenerative diseases.

Globalisation, medical advances, technological developments, urbanisation, migration, and socioeconomic statuses in the 20th century have greatly influenced the global health status. Contrary to this, demographic projections in the 21st century suggest that age-related diseases pose a fundamental threat to the global health system and the health status of the elderly. The mechanism of ageing is complex and not fully understood. However, cumulative oxidative stress and chronic inflammation are the main features that have been theorised to play an essential role in age progression and chronic degenerative diseases [6]. Numerous normal cell metabolic processes in the human body (digesting food, breathing, alcohol and drug metabolism), besides genetic or environmental factors such as air pollutants, cigarette smoking, toxins, and radiation, generate toxic compounds called free radicals. Free radicals are oxygen-containing ions, molecules, or atoms with one or more unpaired electrons in the outermost orbit (valence shell) [7]. These molecular species are highly reactive, unstable, and capable of existing independently, thus, harmfully modifying deoxyribonucleic acid (DNA), proteins, and lipids and triggering several types of human diseases (Figure 1).

Patel [8] defined oxidative stress as “an imbalance between pro-oxidants and antioxidants with concomitant redox circuitry disruption and macromolecular damage.” Oxidative stress contributes to human skin ageing and dermal impairment and is ordinarily regarded as a pivotal contributor to the aetiology and pathogenesis of several chronic diseases [3,4,5]. Reactive oxygen species (ROS), reactive nitrogen species (RNS), and reactive lipid species all constitute the reactive species generated by pathological oxidative stress. Reactive oxygen and reactive nitrogen species (RONS) are comprised of unstable free radicals such as hydroxyl (OH^•^), superoxide anions (O_2_^•−^), nitric oxide (NO^•^) radicals, and non-free radicals, such as peroxynitrite (ONOO^−^) and hydrogen peroxide (H_2_O_2_). The cell mitochondria are responsible for generating the majority of intracellular ROS through oxidative phosphorylation (OXPHOS) [9]. The two predominant systems in ROS production include the mitochondrial OXPHOS and the nicotinamide adenine dinucleotide phosphate- (NADPH^−^) oxidase (NOX) system. The primary endogenous source of ROS is generated from the mitochondrial electron transport chain (ETC) during OXPHOS. During this procedure, O_2_ is degraded to form H_2_O. Other sources implicated in ROS production include immune cells (macrophages and neutrophils), which generate ROS based on the NOX2 isoform as a result of their oxygen-dependent technique to battle against foreign invaders, and peroxisomes and microsomes, which are the primary source of H_2_O_2_ [7]. At low concentrations, ROS plays a critical role in cellular signalling and regulation of processes involved in sustaining homeostasis. ROS, generated by specific plasma membrane oxidases in response to cytokines and growth factors, function as secondary messengers for specific signalling cascades to trigger physiological changes such as gene expression. Intracellular ROS concentration plays a vital role in various cellular processes such as cellular apoptosis, the activation of transcription factors, and the phosphorylation of proteins [10]. Elevated ROS compromises cellular function and activates mitogen-activated protein kinases (p38) (p38MAPK) for p16 upregulation, resulting in cell senescence and the advancement of ageing and chronic degenerative diseases [11]. Elevated ROS formation stimulates aberrant cellular proliferation, unrestrained cell growth, and apoptosis.

Inflammation signifies the innate immune system’s defensive and adaptative response against an injury and/or harmful objects (such as bacteria, viruses, and toxins) to re-establish homeostasis. The inflammatory cascade displays both the advancement and the evolution of the disease and stimulates disease progression. The apt regulation of the inflammatory cascade is crucial for avoiding damage to healthy cells. A dysregulated inflammatory response activates further inflammatory responses, which can cause sepsis and organ failure. Chronic inflammation can occur due to pro-inflammatory cytokine secretion, stimulated by senescent cells [12,13]. The phenomenon of low-grade chronic inflammation is characteristic of human ageing and is termed “inflammaging”. Consequently, the inflammatory cascade elevates extracellular ROS concentrations and oxidative stress. Elevated oxidative stress can result in the breakdown of the extracellular matrix (ECM) and activation of cell necrosis and apoptosis. The presence of necrotic cells and impaired ECM emits numerous constituents that over-activate the inflammatory pathway, resulting in a concatenation of events involving increased production of free radicals and oxidative stress [14,15].

**Figure 2:**
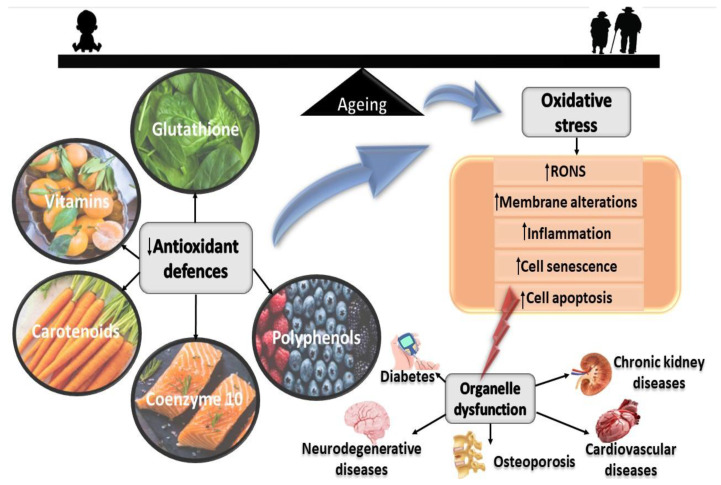
Influence of oxidative stress and the interface of ageing and chronic degenerative diseases. The accretion of oxidative stress and the defective antioxidant defence system contributes to elevated membrane alterations, inflammation, cell senescence, and cell apoptosis. This may subsequently contribute to chronic degenerative diseases.

To counteract or neutralise the effects of free radicals, the human body generates antioxidants. The stability of free radicals and antioxidants is vital for the appropriate physiological function of the body. Oxidative stress develops due to low antioxidant levels and the disruption of the dynamic redox circuitry system, resulting in the accretion of free radicals in the body. Oxidative stress triggers an adverse chain reaction, resulting in the alteration of the cell chemical structure, destruction of the cell membrane, blockage of the cells’ main enzyme actions and energy generation, and prevention of cellular processes essential to the normal and daily functioning of the body [16]. Antioxidants significantly delay or prevent cellular damage and provide pivotal protection against oxidative stress. Some of the significant enzymatic antioxidants include superoxide dismutase (SOD), glutathione peroxidase (GPX), catalase (CAT), and thioredoxin (Trx). SOD catalyses the conversion of O_2_^•−^ to the less reactive H_2_O_2_. GPX catalyses the decomposition of H_2_O_2_ and lipid hydroperoxide (LOOH), while CAT catalyses the oxidation of H_2_O_2_ to H_2_O and molecular oxygen (O_2_), and Trx catalyses the conversion of H_2_O_2_ to produce H_2_O [17]. The most commonly known non-enzymatic antioxidants are lipophilic, including carotenoids, ubiquinol and alpha-tocopherol, and hydrophilic vitamin C, bilirubin, uric acid, albumin, and flavonoids. Elevated RONS and debilitated antioxidant defence systems can advance the progression of ageing and chronic degenerative diseases (Figure 2).

One of the therapeutic remedies for age-related and chronic degenerative diseases is stem cell intervention. Stem cell transplantations counteract immunosenescence through replacement, reprogramming, and restoration [18]. Understanding the mechanisms of oxidative stress concerning chronic inflammation and their role in developing age-related and chronic degenerative diseases is crucial for the invention of therapeutic methods and the management of chronic degenerative complications.

## 2. Molecular Connectivity of Oxidative Stress-Induced Diseases

Inflammaging is characterised by chronic, low-grade inflammation and persistent secretion of proinflammatory cytokines that modify cellular function, even in the absence of overt infection. Furthermore, it increases the presence of inflammatory cells, such as neutrophils, monocytes and macrophages, and C-reactive protein (CRP). Ageing alters the function of the immune system, and in the process of inflammaging, innate immunity establishes minimal changes in mild hyperactivity of circulating inflammatory factors, whereas the adaptive immunity shows a decline effect, and with the progression of adaptive immunosenescence, anti-inflammatory mechanisms are inadequate and impotent to avert the improperly active innate immunity. Chronic inflammation is associated with numerous age-related and chronic degenerative diseases, namely AD, PD, CVD, dementia, osteoporosis, diabetes, and cancer [19,20].

### 2.1. Oxidative Stress and Erythrocytes

Human erythrocytes or red blood cells (RBCs) have an average lifespan of 120 days in vivo. Hematopoietic stem cells undergo differentiation in the bone marrow to generate nucleate erythrocytes. To generate mature, non-nucleate, disc-shaped, biconcave erythrocytes, ribosomes and organelles such as the endoplasmic reticulum (ER) undergo degradation in reticulocytes, and the plasma membrane is remodelled [21]. The primary function of haemoglobin (Hb), a conjugate protein found in high concentrations in erythrocytes, is the transportation of respiratory gases between tissues. Hb also plays an essential role as a source of O_2_^•−^ generation in erythrocytes. Interfaces between oxygenated Hb and heme iron results in an electron transfer [22]. Hb auto-oxidises during this exchange, resulting in methemoglobinemia (MetHb) and O_2_^•−^ generation [22].

Mature erythrocytes preserve a range of proteins, carbohydrates, enzymes, anions, lipids, and cations, and all of these must be balanced for implicit metabolism and cellular function. A key consequence of erythrocyte component imbalance is a decreased ability to cope with oxidative stress. This can lead to degenerative changes in Hb, enzymes, and cell membranes, essential for optimal erythrocyte function [23]. 

Erythrocytes are essential in various metabolic and physiologic activities. As oxygen transporters, erythrocytes are one of the first cells to undergo distress due to exposure to a wide array of adverse environments. Erythrocytes are constantly exposed to high oxygen tension resulting in irreversible damage caused by oxidative stress, which lowers antioxidant capacity, causing erythrocytes to be damaged by haemolysis and removed from circulation [24]. Furthermore, erythrocytes cannot restore degenerative components since they lack intracellular organelles resulting in limited metabolism. According to Abdallah et al. [25], the polyunsaturated fatty acids (PUFAs) profile of the erythrocytes’ plasma membrane and the unremitting exposure to circulating ROS renders erythrocytes highly susceptible to oxidative damage. Thus, elevated oxidative stress shortens the survival of circulating erythrocytes [26]. Under pathological conditions, such as CVDs, diabetes, and ageing, there is an elevation in the frequency of oxidative damage to erythrocytes. Celedón and colleagues [27] demonstrated that biochemical modifications resulting from acute hypobaric hypoxia make erythrocytes susceptible to oxidative stress. During the natural lifespan of erythrocytes, there are numerous changes in size and lipid and protein content in the plasma membrane, such as shrinkage in erythrocyte volume, with an increase in cell density and a decline in Hb content [28]. These changes are consistent with the expulsion of plasma membrane components, such as phospholipids, cholesterol, and integral proteins, including band-3 proteins (B3p) and glycophorin, resulting in a decline in the cell’s surface area [29]. B3p are polytopic membrane proteins, which function as facilitators in the cellular exchange of bicarbonate (HCO3^−^) with chloride ions (Cl^−^) in the plasma, referred to as the “chloride shift” [30]. Therefore, increased oxidative stress alters erythrocyte morphology, elevating susceptibility to the mechanic and osmotic shock and modifying the anion exchange process mediated by B3p [31].

### 2.2. ROS and Ageing

Ageing is the progressive or sequential loss of the tissue and organ anatomical and structural function, which may result in general debility and death. Although ageing is not an explicit disease entity, it is associated with a myriad of chronic disorders. The ‘free radical theory of ageing’ is based on the hypothesis that ROS is the cause of structural damage and functional losses due to the accumulation of oxidative damage to cell constituents and connective tissues [32]. The ageing process can occur as a result of intrinsic factors (genetics) or extrinsic factors (improper diet, smoking, lack of physical activity, substance abuse, and untreated inflammatory conditions). Literature shows that numerous deleterious mechanisms, including mitochondrial dysfunction, the accumulation of oxidative damage, genomic instability, loss of proteostasis, stem cell exhaustion, and cellular senescence, are correlated with ageing [33]. Presently, the free radical theory and the mitochondrial theory are two acknowledged theories on the mechanism of ageing. The ageing mechanism is based on the hypothesis that elevated concentrations of intracellular free radicals induce mitochondrial dysfunction and modify cellular structural function and regeneration. The mitochondria and the NOX system are the prime players involved in excessively generating cellular oxidative stress. Egea et al. [34], Park et al. [35], and Zhang et al. [36] demonstrated that chronic degenerative diseases exhibited increased expression and/or activity of NOX. There has been speculation that elevated RONS concentrations and oxidative stress induce cellular senescence, which is characterised by the cessation of cellular proliferation in normal and pathophysiological processes. Moreover, senescence-associated secretory phenotype (SASP) is provoked by multiple stimuli, such as the secretion of degenerative matrix metalloproteases (MMPs), insoluble extracellular matrix (ECM) components, and soluble chemokines, cytokines, and growth factors [37].

### 2.3. Neurodegenerative Diseases 

#### 2.3.1. Alzheimer’s Disease (AD)

AD is one of the most typical neurodegenerative diseases that affect individuals with advanced age. It is illustrated by the continuous loss of neuronal function and cognitive impairment, deposition of Tau proteins forming neurofibrillary tangles (NFT), and the formation of amyloid-beta (Aβ) plaques. Under normal physiological concentrations, Aβ modulates neural growth and repair and plays an essential role as a natural antioxidant [38]. The evolution and advancement of AD are thought to be induced by oxidative imbalance. According to Menzies et al. [39], impaired neuronal mitochondrial metabolism decreases adenosine triphosphate (ATP) generation, elevates O_2_ free radicals, and increases the generation of extracellular Aβ and the phosphorylation of intracellular Tau proteins. The precise mechanisms underlying the imbalanced redox state in AD remain unknown; however, excessive oxidative stress was discovered in the infancy of AD, preceding the accumulation of marked Aβ [40].

The accumulation of Aβ and the hyperphosphorylation of Tau proteins elevates ROS generation by stimulating the c-Jun N-terminal kinase (JNK)/p38 MAPK signalling cascades. Studies conducted by Caspersen et al. [41], and Manczak et al. [42], found that brain mitochondria of transgenic mice, AD patients, and neuroblastoma cells expressing human mutant amyloid precursor protein (APP) contained Aβ deposits due to deficient antioxidants. This process exacerbates mitochondrial ROS generation and induces mitochondrial dysfunction. It is considered that the presence of Aβ deposits may have destructive effects on mitochondrial metabolism, leading to mitochondrial dysfunction and neuronal death. Additionally, in the ER, the aggregation of Aβ leads to an imbalance in calcium (Ca^2+)^ homeostasis [20]. The orifice of mitochondrial permeability transition pores, N-methyl-4-phenyl-1,2,3,6-tetrahydropyridine (MPTP), and mitochondrial-generated ROS induce the accumulation of mitochondrial Ca^2+^ [43]. Cellular Ca^2+^ overload under pathological conditions is primarily associated with oxidative stress, and mitochondrial Ca^2+^ uptake may initiate neurotic states and cell death. In combination with NO^•^, the uptake of mitochondrial Ca^2+^ can spark the mitochondrial membrane’s disintegration and hasten cell death.

The interaction between the metal ions and the Aβ plaques is believed to be involved in the generation of H_2_O_2_. Riederer et al. [44] reported elevated concentrations of metal ions such as zinc, iron, and copper in Aβ plaques. According to Wang et al. [40] and Zhao and Zhao [45], Aβ-induced oxidative damage elevates the formation of by-products associated with DNA/RNA, lipid, and protein oxidation. With the progression of AD, there is a decline in the deposition of Aβ due to ROS-induced oxidative imbalance. Furthermore, there is a decline in antioxidant enzymes, such as SOD and CAT, as well as vitamin E and C. Individuals with AD demonstrate a decline in the actions of pivotal oxidative enzymes such as cytochrome oxidase and pyruvate dehydrogenase and α-ketoglutarate dehydrogenase complexes [40].

The nuclear factor erythroid 2-related factor 2 (Nrf2) pathway has an enormous influence on AD, and is an evolving therapeutic target of AD. It is an essential redox-regulated transcription factor that is critical in regulating oxidative stress-related genes. According to Bahn and Jo [46], neurodegenerative disorders such as AD contain impaired function and altered localisation of Nrf2. Nrf2 functions as an upregulator of antioxidative defence, impeding inflammatory responses and preserving proteostasis. In physiological conditions, kelch ECH associating protein 1 (KEAP1), a constituent of the cullin3-based E3 ligase complex, cloisters Nrf2 in the cytoplasm. This results in the poly-ubiquitination of Nrf2 and successive Nrf2 degradation by the ubiquitin-proteasome cascade [47]. On the contrary, the KEAP1/Nrf2 interface is interrupted by Nrf2 and ROS activators reacting with KEAP1 cysteine residues [47]. This results in the alterations of KEAP1 cysteine residues and subsequent deterioration of the KEAP1 ubiquitin ligase activity [48]. The accumulation of Nrf2 in the nucleus follows after Nrf2 degradation undergoes suppression and is stabilised. This leads to an interface between Nrf2 and binding elements, which spearheads detoxification and antioxidant gene transcription [49]. Therefore, the KEAP1-Nrf2 cascade regulates detoxification and antioxidant genes responsible for the fortification of cells from electrophilic and oxidative stress. A study conducted by Kubben et al. [50] demonstrated that suppression of the Nrf2 signalling cascade promotes the premature ageing phenotype of Hutchinson–Gilford progeria syndrome (HGPS). They further determined that reactivation of the Nrf2 signalling cascade leads to a decline in ROS generation and repairs cellular HGPS deficiencies. Furthermore, Uruno and colleagues [48] determined that the induction of Nrf2 in App^NLGF^ model mice enhanced antioxidative properties in the brain, thus improving pathological neuroinflammatory responses. Their study suggested the kEAP1-Nrf2 regulatory cascade as a potential therapeutic target for advancing drugs affecting neurocognitive pathologies, including AD.

#### 2.3.2. Parkinson’s Disease (PD)

PD is a progressive neurodegenerative disease caused by the degeneration of dopaminergic neurons and an abnormal increase of α-synuclein (α-syn) within the substantia nigra (SN; darker appearing areas in the brain as a result of high levels of melanin in dopaminergic neurons). It is believed that the involvement of dopamine (DA), Ca^2+^, iron, neuroinflammation, and mitochondria critically contribute to increased oxidative stress and neurodegeneration. Disrupted redox potential in neurons obstructs several biological progressions resulting in cellular death, and ROS generation plays a significant role in the progression of PD [20]. In PD, the discovery of the neurotoxin, 1-methyl-4-phenyl-1,2,3,6-tetrahydropyridine (MPTP), was initially linked to mitochondrial dysfunction and loss of SN [51]. PD is typified by the loss of dopaminergic neurons. Bindoff et al. [52] reported that the SN of individuals with PD displayed reduced complex I of the mitochondrial ETC and ubiquinone, leading to neurodegeneration. In addition, MPTP, paraquat, and rotenone elevate ROS production, thus causing the progressive loss of dopaminergic neurons. 1-methyl-4-phenylpyridinium (MPP^+^), a toxic MPTP metabolite, is formed when MPTP crosses the blood–brain barrier and is metabolised by astrocytes [53]. The accumulation of MPP+ constrains the function of complex I, disrupting electron translocation via the mitochondrial ETC. This leads to a reduction in the production of ATP and elevated generation of ROS, initiating Parkinsonism.

Tyrosine hydroxylase (TH) is an enzyme involved in converting the amino acid tyrosine to DA, a neuromodulatory molecule formed by dopaminergic neurons, essential for motor activity. Oxidative stress is speculated to be involved in the degeneration of dopaminergic neurons. In PD, l-3,4-dihydroxyphenylalanine (L-DOPA), as a precursor to DA, is involved in the synthesis of DA in the presence of DOPA decarboxylase (DDC) [54]. Following its synthesis, DA is transferred to a stable environment and stored in synaptic vesicles within the cytoplasm and is dependent on the uptake of DA by the vesicular monoamine transporter 2 (VMAT2). TH, DDC, and VMAT2 form a complex that averts DA from being released into the cytosol, thus facilitating its storage within the synaptic vesicles [54]. Damaged neurons have an excess quantity of cytosolic DA due to the reuptake of impaired DA in the extracellular space of the synaptic vesicle, which undergoes auto-oxidation or enzymatic metabolism by monoamine oxidase (MAO), yielding H_2_O_2_ as a by-product [55]. DA quinones (DAQ) or semiquinones are vastly reactive oxidized DA and are generated due to O_2_^•−^ radical reduction during DA oxidation [56]. DAQ promotes neuronal degeneration and induces modification of PD-related proteins, namely: α-syn, SOD-2, parkin, ubiquitin C-terminal hydrolase L1 (UCH-L1), and DJ-1 [57]. In addition, DAQ is responsible for mitochondrial dysfunction and inactivation of the TH enzyme and the DA transporter (DAT). The formation of neuromelanin occurs due to the oxidation of DAQ to aminochrome, generating O_2_^•−^ radicals and degradation of cellular NOX. Neuromelanin, a catecholamine-based polymer pigment, aggregates in the human brain’s SN pars compacta (SNpc) [58]. The gradual loss of DA neurons in the SNpc and the intracellular sedimentation of misfolded α-syn are associated with the pathogenesis of PD.

The inflammatory response of the nervous system, neuroinflammation, restores and protects the anatomical structure and function of the central nervous system (CNS) against traumatic insults and damage, toxic metabolites, autoimmunity, and infectious agents. It is characterised by the activation of microglia [59]. Microglia are innate immune cells of the brain that play a pivotal role in immune defence and modulating brain development. Microglial activation releases numerous neurotoxicants such as proinflammatory cytokines, such as interferon gamma (IFN-γ), IL-1β, IL-2, IL-6, and tumour necrosis factor-alpha (TNF-α), and mediators of inflammation, namely COX-2 and inducible nitric oxide synthase (iNOS) [60]. The continuous release of inflammatory mediators and proinflammatory cytokines induces NO^•^ and O_2_^•−^, contributing to oxidative stress and RNS in the CNS. Consequently, this process may lead to the development of chronic inflammation. According to Calabrese et al. [61] and Pal et al. [62], PD patients display many activated microglia and elevated concentrations of neuroinflammatory markers, including IFN-γ, IL-1β, IL-6, and TNF-α. Managing microglia activation may be essential in reducing high levels of ROS production and PD pathogenesis.

### 2.4. Cardiovascular Diseases (CVDs)

CVDs are a leading cause of global mortality and morbidity and are a principal contributor to disability in the elderly. Hypertension and hypercholesterolemia have been implicated as the main risk factors that augment ROS production and the development of oxidative stress [63]. Oxidative stress plays a vital role in the evolution and advancement of CVDs, including altering gene expression. Studies conducted by Bulua et al. [64] and Zhou et al. [65] indicate the pivotal role played by oxidative stress in facilitating cytokine generation and secretion and interconnecting ROS with vascular endothelial activation, dysfunction, and inflammation. Vascular endothelium is primarily responsible for the generation of NO^•^, which is critical in the modulation of blood pressure and vascular tone and optimal performance of the heart and vascular system. Essentially, NO^•^ is a crucial molecule required in numerous cell processes, including maintaining vascular homeostasis in endothelial cells.

As a result of ROS’ dual faceted mechanism in cardiovascular pathophysiology, at low concentrations, it bestows a remarkable contribution to the benefit of the cardiovascular system, such as endogenous cardiovascular protective, pro-angiogenesis, and anti-atherosclerotic effects. High concentrations of ROS induce a variety of disorders by stimulating endothelium-derived contracting factors (EDCFs) and creating atherosclerosis, and the reduction of NO^•^ bioavailability marks the inception of endothelial dysfunction [66]. The formation of ONOO^−^ occurs when O_2_^•−^ reacts with NO^•^, and the successive generation of ONOO^−^ induces endothelial cell dysfunction and death. According to Elahi et al. [67], elevated ROS concentrations regulate the activity of transcription factors, namely, activator protein 1 (AP-1), nuclear factor-kappa B (NF-κB), and the peroxisome proliferators-activated receptor (PPAR) family.

One of the initial causal events of atherogenesis or other CVDs associated with endothelial dysfunction is low-density lipoprotein (LDL) oxidation within the vessel wall [68]. Atherosclerosis, at locations of disrupted flow patterns, is initiated when oxidised low-density lipoprotein (oxLDL) is transferred to the tunica media from the vessel lumen. Oxidised phospholipids, produced in proinflammatory tissues such as atherosclerotic abrasions through receptor-independent or receptor-mediated signalling reactions, elevate proinflammatory gene activity and growth factors, signal monocytes, stimulate endothelial cells, produce endothelium adhesion molecules expression, and possess endothelium cytotoxic effects [69]. Dose-dependent elevations in ROS generation cause oxLDL to transfigure the intracellular redox state of a cell by binding to the endothelial lectin-like oxLDL receptor-1 (LOX-1) [70]. Paik and colleagues [71] conducted a study on 2944 healthy women aged between 30 and 79 years to explicate the effect of age on the atherogenicity of inflammatory markers and LDL. The results highlighted elevated oxLDL levels in the plasma after 50 years. Cominacini et al. [72] stated that the upregulation of ICAM-1 and VCAM-1 is activated by oxLDL. This action is further magnified by cytokines such as vascular endothelial growth factor (VEGF), TNF-α, angiotensin II (Ang II), and interleukins, which stimulate vascular NOX to excessively generate ROS. Results from a study conducted by Touyz and Schiffrin [73] suggested that Ang II-elevated NOX-ROS production in smooth muscle cells and induced vascular remodelling in hypertension. In another study, Touyz and Schiffrin [74] demonstrated that the action induced by Ang II on the Ang II type 1 (AT1) receptor activates protein kinase C (PKC). PKC activation is responsible for the inception of ROS generation, which leads to Src kinase and cellular Src tyrosine kinase stimulation. Additionally, oxLDL contributes to NF-κB activity primarily observed in atherosclerosis. The ROS/p38MAPK/NF-κB pathway is employed by oxLDL to stimulate the expression of cell adhesion molecules (CAMs) and monocyte-endothelial adhesion [75]. Under abnormal physiological conditions, all the layers of the blood vessel wall can generate ROS, most of which are resultant of NOX enzymes. As a result of elevated ROS concentrations, NO^•^ bioavailability is reduced, leading to a decline in endothelium-dependent relaxation. Intriguingly, a study conducted by Stielow and colleagues [76] displayed that the novel NOX inhibitor VAS2870 inhibits oxLDL-mediated O_2_^•−^ formation from human endothelial cells.

### 2.5. Diabetes

One of the reasons for the increased rate of DM in the aged population is increased insulin resistance with age due to sarcopenia, obesity, and lessened physical activity besides general health status, including the presence of frailty and comorbidity. Diabetes develops when blood glucose is overly high (hyperglycaemia). Chronic hyperglycaemia is linked to the progression of DM complications due to altered signalling pathways, oxidative stress, advanced glycation end products (AGEs), and the secretion of the proinflammatory cytokines and cellular apoptosis. Furthermore, hyperglycaemia triggers the formation of diacylglycerol (DAG) and, through the activation of the PKC pathway and NOX, advances the production of ROS and oxidative stress. For type II diabetes, the reduction of glucose absorption into adipose and muscle tissue induces chronic hyperglycaemia. As a result, tissue damage and abnormal physiological conditions (involving atherosclerosis, heart disease, and retinopathy) ensue [77]. Primarily, diabetic patients develop microvascular and macrovascular complications, which form part of the principal sources of disability and mortality. In type II diabetes, it is suggested that exposure of the pancreatic β cells to ROS and oxidative stress leads to the development of defective β cells, which are unable to produce and/or release sufficient insulin [18]. RONS exhibit a bidirectional modulation in insulin signalling. It is now crescively evident that RONS acts as inhibitors of the insulin signalling pathway, rendering them putative mediators in the evolution of insulin resistance. However, RONS acts as a facilitator of this pathway, ensuring that the cellular and physiological effects of insulin are exerted. Elevated oxidative stress can be directly induced by glucose oscillations, which are fundamental in altering the primary culprit of diabetes [78,79].

There are four fundamental theories highlighting the role of hyperglycaemia as the causative agent in diabetic complications: (i) activation of PKC isoforms, (ii) elevated hexosamine cascade flux, (iii) elevated polyol cascade formation, and (iv) elevated formation and glycation of proteins [80]. The interaction between AGEs and receptors for advanced glycation end-products (RAGEs) induces post-receptor signalling and promotes ROS production [77]. In addition, AGEs activate the transcription regulator, NF-kB, which stimulates the transcription of ICAM-1 and VCAM-1, and induces sorbitol, PKC and ROS generation. This demonstrates a hyperglycaemia-mediated mechanism of excessive O_2_^•−^ generation by the mitochondrial ETC. Furthermore, mitochondrial dysfunction reduces ATP production capability, which stimulates the NOX complex, Ca^2+^ signalling, and β cell glucose-stimulated insulin secretion (GSIS) [81]. Additionally, O_2_^•−^ generation can also be attributed to an increase in glycolytic flux, which endorses oxidative phosphorylation and ATP generation. Essentially, the pentose phosphate cascade regulates the inceptive adaptive response, in which glucose carbon diverges excessive glycolysis and oxidative phosphorylation by converting excess glucose to a pentose [82]. However, this process can also lead to elevated O_2_^•−^ synthesis and NOX activity.

NOX is activated in response to elevated levels of AGEs and glucose autoxidation. Pérez-Matute et al. [77] suggested that NOXs role in stimulating basal ROS generation upregulates antioxidant enzyme defences. NOX acts as a double-edged sword, where protracted NOX activations lead to defective antioxidant defences, mitochondrial dysfunction, endothelial NO synthase (eNOS) uncoupling, and induction of oxidative stress. In response to hyperglycaemia, insulin is released into the blood circulation by pancreatic β cells, and its anabolic effect on target tissues is influenced by its transmembrane receptor, insulin receptor (IR) [82]. This interaction promotes the phosphorylation of insulin receptor substrate (IRS) proteins, autophosphorylation of IR, and triggers signalling pathways, such as protein kinase B (Akt) and phosphatidylinositol-3-kinase (PI3K). In addition, intracellular insulin elevation occurs due to increased cellular O-GlcNAcylation, which simultaneously maintains glucose-stimulated insulin secretion in β-cells [83]. This is partly because increased O-GlcNAcylation elevates histone H3 transcriptional activation markers, leading to increased mRNA expression of the insulin (Ins1/2) gene [83]. It has been suggested that O-GlcNAcylation mainly regulates cellular processes, such as transcription, translation, and signal transduction cascades, in response to stress and nutrients [84,85]. Furthermore, O-linked β-N-acetylglucosamine (O-GlcNAc) is a potent post-translational modification on a myriad of proteins directly at or located proximal to serine or threonine residues [86]. O-GlcNAc alters the IRS proteins at specific sites, leading to elevated GlcNAcylation of IRS proteins, which decreases its binding to PI3K p85 regulatory subunit, thus resulting in O-GlcNAc’s downregulation of insulin signalling [87]. A study conducted by Yoon et al. [88] determined that O-GlcNAcase (OGA), a beta-exo-N-acetylhexosaminidase responsible for elevating O-GlcNAc concentrations in cells, and inhibition by PUGNAc ((phenylcarbamoyl) oxime analogue of GlcNAc; molecular weight, 353.3) significantly reduced ROS generation and oxidative-induced loss of mitochondrial membrane potential. According to Henriksen et al. [89], Akt plays a vital role as a regulator of lipid and glucose metabolism and modulates vesicle translocation of glucose transporter 4 (GLUT-4) in insulin-responsive tissues. While NOX can be transiently activated with increased ROS generation, it can also be triggered by the redox-sensitive KEAP1-Nrf2 and receptor tyrosine kinase signalling cascades [90].

### 2.6. Chronic Kidney Disease (CKD)

CKD is common in the elderly, mainly due to the increasing prevalence of diabetes, hypertension, and CVD. CKD is characterised by a gradual decline in kidney function or a glomerular filtration rate (GFR) < 60 mL/min/1.73 m^2^ for the course of 3 months or more, irrespective of the underlying conditions [91]. Potential complications from CKD include CVD, acute kidney injury, anaemia, kidney disease progression, mineral and bone disorders, and cognitive decline.

The kidney is one of the most energy-demanding body organs, with the energy required to maintain renal tubular transport by secreting and reabsorbing substances. Literature indicates that high intracellular levels of ROS play a vital role in the development of CKD. In the kidney, cellular mitochondria and NADPH oxidases are the leading causes of ROS production. However, in normal circumstances, the renal antioxidant system, including CAT, GPX, and SOD, corrects any ROS-facilitated injury. Renal injury is characterised by the excessive production of mitochondrial ROS [92]. Specific biomolecules undergo oxidation when there is an imbalance in the redox systems, resulting in anatomical and structural modifications of these molecules. This process occurs in the mitochondria and is orchestrated by mitochondrial cytochrome oxidase enzymes such as cytochrome P450. Su et al. [93] and Zhu et al. [94] conducted studies on aldosterone-infused mouse models, and their findings suggested that mitochondrial dysfunction precedes proteinuria and podocyte effacement. ROS, generated as a by-product during this process, facilitates atherosclerosis pathogenesis in CKD and the development of renal injury.

Ratliff and colleagues [95] suggested that the downregulation of SOD and the overexpression of NOX in CKD indicates a correlation between the accumulation of O_2_^•−^ and oxidative stress in renal failure. In addition, ONOO^−^ is generated when NO^•^ reacts with O_2_^•−^ and gives rise to nitrosative stress, a NO-mediated nitrosylation of redox-sensitive thiols. ONOO^−^ induces oxidative damage and alters cellular signalling cascades by oxidising DNA, lipids, and proteins, inciting cellular injury, necrosis, and apoptosis. Furthermore, myeloperoxidase (MPO) metabolises Cl^−^ and H_2_O_2_ to hypochlorous acid (HOCl), thereby contributing to chlorinated stress [96]. Liu and colleagues [97] and Malle et al. [98] reported on the elevation in HOCl-mediated protein oxidation in kidney tissues of individuals with CKD. Additionally, studies conducted by Nicholls et al. [99] and Xu et al. [100] demonstrated that MPO-mediated HOCl uncouples and impedes eNOS and impairs high-density lipoproteins (HDL) in atherosclerotic lesions. Carbonyl stress is induced due to the elevated generation of AGEs in renal dysfunction, which further stimulates inflammation in CKD [96]. Moreover, leukocyte recruitment and activation and the generation of AGEs, oxLDL, and advanced protein oxidation products are induced by oxidative stress [101]. The activation of immune cells, such as neutrophils and macrophages, and resident cells, prolongs the oxidative state due to the production of RONS. The excessive generation of mitochondrial ROS promotes CKD-mediated chronic micro-inflammation by stimulating the NOD-, LRR-, and pyrin domain-containing protein 3 (NLRP3) inflammasome in patients with uraemia [102].

Factors such as angiotensin II decreased NO^•^ generation, and hypertension further promotes the surge of ROS generation in CKD. Studies conducted by An et al. [103], Cho et al. [104], and Quiroz et al. [105] demonstrate the impact of ROS generation in the advancement of CKD, in which antioxidants such as omega-3 fatty acids, niacin, and melatonin, mitigate kidney injury.

## 3. Stem Cell Interventions

In ageing, stem cells become defunct and are associated with the deterioration of physical and mental capabilities [106]. In recent years, stem cell applications utilised human neural stem cells (hNSCs) to replace damaged neutral structures (Table 1). The rationale therapy in NSC applications involves replacing lost neurons, regulating disordered neurotransmission, and restoring functional activity. NSCs are a group of ectodermal progenitor cells with the capability to self-renew and differentiate into specialised neutral subtypes, including glial cells and neurons [107,108]. Consequently, NSCs display an inherent mechanism to rescue dysfunctional neural pathways and are an appealing, universal source for grafting and the advancement of restorative cell therapies.

Moreover, NSC therapy plays a critical role in replacing DA-producing neurons in PD, and in support of this, Trounson et al. [109] highlighted numerous clinical and preclinical trials. A study conducted by Zuo and colleagues [110] to assess the potential effects of hNSCs on PD found that hNSCs effectively restored and enhanced the functional defects in intrastriatal 6-hydroxydopamine-induced (6-OHDA) Parkinsonian mice. In another study, Lévesque et al. [111] demonstrated that autologous NSCs produced motor improvement and elevated DA uptake. Limitations observed with NSC therapy in treating CNS pathologies include deficient functional recovery due to the implanted NSCs’ inability to connect with existing neurons. Additionally, failure of the intravenously injected NSCs to travel through the lungs results in a small quantity of NSCs accessing their target regions in the brain [112]. Mesenchymal stem cells (MSCs) from juvenile animals play a pivotal role in the phenomenon of stem cell exhaustion and offer an excellent promise for delaying chronic degenerative pathologies. Essentially, MSC therapy has arisen as a potential contender for treating CNS pathologies. Literature indicates that stem cells regulate physiological homeostatic control [33,113].

**Table 1:** NSC studies in chronic degenerative diseases.

	Cell Line	Studied Model	Tested Parameters	Observations	Ref.
**Alzheimer’s disease**	NSCs	Aged triple transgenic mice (3xTg-AD)	Cognitive function and behavioural tests. Migration and differentiation of engrafted cells.	Differentiation of NSC into astrocytes, neurons, and oligodendrocytes. NSC liberates spatial learning and memory deficits. Improved cognitive function, mediated by elevated BDNF, and elevated hippocampal synaptic density.	[114]
	NSCs	Sprague-Dawley rats	Spatial cognitive capability. Neuronal migration, differentiation, and survival of engrafted cells.	NSCs differentiation into neurons and glial cells. Significant increase in cholinergic neurons of NSCs-transplanted group. Significant statistical improvement in the spatial cognitive capability of NSCs-transplanted group.	[115]
	NSCs	Rats with fimbria-fornix lesions	Neuronal differentiation and survival in the hippocampus and basal forebrain. Functional effects.	Cells differentiated into neurons and glial cells. Differentiated cells acquired neuron-like features, as well as neurofilament subunit expression. Enhanced survival of NSC. Increase in cholinergic neuronal phenotype, with enhanced expression of the p75 neurotrophin receptor and choline acetyltransferase.	[116]
	NSCs	Sprague-Dawley rats	Neuronal differentiation and survival. Memory and learning abilities.	Cells differentiated into neurons and glial cells. Significant increase in the expression of p75 neurotrophin receptor. BDNF improved the treatment effects NSCs transplanted group.	[117]
**Parkinson’s disease**	NSCs	6-OHDA- lesioned Sprague-Dawley rats	Behavioural benefits/testing. Protection against dopaminergic exhaustion.	Significantly improved parkinsonian symptoms. Preservation of TH. NSC transplantation exerted neuroprotective properties against dopaminergic exhaustion as a result of neuronal differentiation and the secretion of tropic factors.	[118]
	NSCs	6-OHDA- lesioned rats	Cell survival and migration to Striatum. Neuronal differentiation.	Improved cellular migration over the striatum. Expression of DA-synthesising enzymes, TH, and L-amino decarboxylase.	[119]
	ESCs	6-OHDA- lesioned Sprague-Dawley rats	Cellular proliferation and differentiation. Functional recovery.	Proliferation of ESCs into fully differentiated DA neurons. Persistent behavioural restoration of DA-induced motor asymmetry.	[120]
**Diabetes**	NSCs	Sprague-Dawley rats with DR	BDNF and Thy-1 expressions. DR progression.	NSC transplantation reduced retinal vascular dysfunction. Significant increase in BDNF and Thy-1 expressions. Elevated number of surviving RGCs. Significantly diminished DR progression.	[121]
**Hypoxic-ischaemic injury**	NSCs	Sprague-Dawley rats with neonatal HI	Motor behavioural tests. Axonal sprouting, neuronal differentiation, and microglia response.	Enhanced motor function recovery. NSCs grafts demonstrated good survival and differentiation and modified microglial response. Enhanced axonal sprouting. Upregulation of neurogenesis, neurotrophic and gliogenesis genes.	[122]
	NSCs	Sprague-Dawley rats	VEGF protein expression and neuronal apoptosis.	Diminished neuronal apoptosis. Elevated angiogenesis.	[123]
	NSCs	Sprague-Dawley rats	Neurological outcomes.	Enhanced sensorimotor function. Diminished brain tissue loss. Inflammation suppression.	[124]

NSCs, neural stem cells; BDNF, brain-derived neurotrophic factor; TH, tyrosine hydroxylase; 6-OHDA, 6-hydroxydopamine; Thy-1, thymocyte differentiation antigen 1; RGCs, retinal ganglion cells; DR, diabetic retinopathy; DA, dopamine; ESCs, embryonic stem cells; HI, hypoxic-ischaemia.

MSCs can be acquired from the adipose tissue, bone marrow, foetal liver, muscle, umbilical cord, and lungs. Clinical and preclinical investigations conducted by Chen et al. [125], Hayashi et al. [126], Nöth et al. [127], Richardson and Hoyland [128], and Tzaribachev et al. [129] suggest that MSCs play a pivotal role in wound repair, including growth and replacement of damaged cells. The expression of chemokine receptors in injured tissue attracts MSCs toward inflammatory regions. MSC therapy is recommended as a potential contender for treating neurodegenerative diseases. Implantation of MSCs in the damaged tissue regions induces therapeutic effects through various mechanisms (Table 2), such as differentiation, anti-inflammatory and immunomodulatory effects [130], neurogenesis induction, and astroglial stimulation [131], a decline in oxidative stress and apoptosis [132], increased axon growth [133], and neurotrophic factor secretion [134]. A study conducted by Kim and colleagues [135] demonstrated that MSCs secreted ICAM-1, reducing Aβ plaques in 10-month-old transgenic mouse models of AD. In another study, Park et al. [136] examined the ameliorative effects of MSCs on neurogenesis in PD models. They reported that human MSCs significantly elevated neurogenesis in the subventricular zone of PD animal models, which led to the differentiation of neural precursor cells into dopaminergic cell groups in the substantia nigra in PD. Despite all of these findings, Peng and colleagues [137] suggested the need for additional data to validate the efficiency of MSC therapy in the treatment of neurodegenerative diseases. Additionally, limitations of MSC therapy include an inefficiency of distribution and survival rates in implanted modalities.

## 4. Antioxidant Defences

Antioxidants suppress oxidative stress-associated destruction by disintegrating radical chain reactions [155]. By freely accepting and/or donating electrons, antioxidants can counteract free radicals by eradicating the free radicals’ unpaired state. This section illustrates antioxidants’ crucial roles in counteracting oxidative stress-induced age-related and degenerative pathologies.

### 4.1. Glutathione

Glutathione is a vital antioxidant present in all cells. It helps protect healthy cells by averting ROS-induced cell damage. Glutathione plays a role in antioxidant defence and electrophilic xenobiotics detoxification through enzymatic reactions involving GPX, glutathione reductase, and glutathione-S transferase [156]. These three groups of enzymes comprise the glutathione cycle, which helps repair ROS-induced cell damage and protect against the excessive production of ROS. Additionally, glutathione modulates redox-induced signal transduction and metabolism of oestrogens, prostaglandin, and leukotriene. Modifications in glutathione concentrations contribute to the dysregulation of deoxyribonucleotide synthesis, cellular proliferation, apoptosis, and immune response [157].

Glutathione serves as an electron donor, reducing cytoplasmic and protein disulphide bonds to cysteines. In this process, glutathione is oxidised to form GPX or glutathione reductase that, through the NADPH-dependent process, can accomplish glutathione regeneration from glutathione disulphide [158].

A deficiency in glutathione homeostasis leads to increased oxidative stress and the development of neurogenerative diseases such as PD, dementia, and AD. In patients with PD, glutathione is decreased by 40–50%, and, depending on the gravity of the disease, the decrease occurs mainly within the brain and the SN. Mischley et al. [159] elucidated that the elevation of ROS production and a decline in glutathione concentration within the midbrain is associated with PD. Chinta and Andersen [160] showed that prolonged glutathione depletion is due to the dithiothreitol- (DTT-) reversible phenomenon entailing cysteine residues, which leads to impairment of mitochondrial complex I subunits which affects its enzymatic activity. In the view that mitochondrial dysfunction impedes dopaminergic neurons, the restoration of glutathione to normal concentrations could provide therapeutic benefits in PD. Furthermore, a reduction in glutathione concentrations in individuals with AD is correlated with the repression of glutathione homeostasis.

Diabetes modifies GPX and glutathione reductase activity. In a study conducted by Martina and colleagues [161], it was suggested that glutathione administration in individuals with type II diabetes enhanced platelet constitutive NOS (cNOS) activity and concurrently decreased plasminogen activator inhibitor-1 (Pal-1). Pal-1 stimulates the dissolution of fibrinolysis, which fundamentally leads to fibrin degradation. Martina and colleagues [161] further suggested that a reduction in glutathione may play a pivotal role in elevated mortality due to CVD in individuals with type II diabetes. Shimizu et al. [162] assessed the correlation between CVD and plasma total glutathione among 134 CVD cases and 435 healthy control subjects. Their data demonstrated a decline in total plasmic glutathione in CVD patients, which is prominent in patients with cerebral haemorrhage and lacunar infarction when compared to their healthy counterparts. This phenomenon suggests that a decline in total plasmic glutathione concentrations is a risk factor for CVD. In a prospective study, Espinola-Klein and colleagues [163] investigated the correlation between GPX-1 activity with atherosclerosis. The findings suggested that GPX-1 activity is associated inversely with CVD risk; that is, an elevation in atherosclerotic vascular beds is accompanied by a decline in GPX-1.

### 4.2. Polyphenols

Polyphenols are specialised metabolites naturally produced by plants and are included in various supplements. These metabolites also occur in large amounts in dietary foods, including fruits, tea, vegetables, and spices. Polyphenols defend against the dissemination of pathogens and ultraviolet damage. Polyphenols are prominent for their health benefits, such as anti-inflammatory, antioxidant, free radical scavengers, and anti-carcinogenic flair [164]. Polyphenols’ limitations are that smidgen concentrations make it to the target organs due to deficient absorption and/or substantial metabolism of the compounds by phase I or II enzymatic reactions [165,166]. Additionally, the ability of phenolic compounds to sustain various modifications before reaching the target organs may crucially influence their aptness properties. Furthermore, polyphenols have been demonstrated to diminish iron-induced DNA destruction by reacting with iron to form a polyphenol-iron complex [167]. Recently, plant polyphenols have created the allure to mitigate oxidative stress as aetiological mechanisms in chronic degenerative diseases, such as CVDs, AD, cancer, and cerebrovascular disease. Additionally, one of the biological effects of dietary polyphenols includes the modulation of gene expression in vascular endothelial cells. Some of the most prevalent classes of polyphenols include flavonoids, stilbenes, phenolic acids, curcuminoids, coumarins, tannins, and lignans. Notably, curcumin has acquired recognition for nutraceutical applications. Curcumin has potent activity as scavengers for ROS such as O_2_^•−^, OH^•^, H_2_O_2_, lipid peroxidases, and numerous RNS [168]. Curcumin also plays a critical role in elevating cellular glutathione concentrations.

#### 4.2.1. Flavonoids

Flavonoids are known for their beneficial effects on both humans and animals. Flavonoids can modulate enzymatic functions and are a principal component in pharmaceutical, nutraceutical, and medicinal applications due to their antioxidative, anti-inflammatory, anti-carcinogenic, and antimutagenic properties [169]. The antioxidant properties of flavonoids include repressing ROS production by inhibiting numerous enzymes and chelating trace elements involved in redox reactions [170,171]. Moreover, flavonoids influence free metal ion levels that encourage ROS generation in the cell by reacting with H_2_O_2_ and forming highly reactive OH^•^ in a chain of Fenton reactions. Besides their inhibitory effect on numerous enzymes such as COX, xanthine oxidase (XO), PI3K, and lipoxygenase, flavonoids upregulate the antioxidant defence system by scavenging ROS [172,173].

Flavonoids directly scavenge free radicals, thereby preventing free-radical-induced injury. Free radicals oxidise flavonoids, thus stabilising the radicals. Flavonoids react with free radicals’ reactive components, thereby stabilising ROS. Due to the shallow redox potential of the OH^•^ groups of flavonoids, highly reactive ROS such as O_2_^•−^, alkoxyl, OH^•^, and peroxyl radicals, are reduced by the hydrogen atom transfer (HAT) mechanism. A study conducted by Hanasaki and colleagues [174] reported on the capabilities of 15 flavonoids to scavenge O_2_^•−^ and OH^•^. Their findings demonstrated that flavonoids such as (−)-epicatechin, (+)-catechin, rutin, and 7,8-dihydroxy flavone are powerful OH^•^ scavengers. Except for monohydroxyl flavones, the flavonoids demonstrated inhibitory effects toward O_2_^•−^ production in the hypoxanthine-xanthine oxidase system. This could be attributed to the suppression of XO activity.

Quercetin (3,3′,4′,5,7-pentahydroxylflavone) is a natural bioactive plant flavonoid found in a variety of derived foods and cultivated plants, including broccoli, black tea, nuts, and grapes, where it forms a quercetin derivative, quercetin glycosides, by amalgamating with residual sugars [175]. In healthy individuals, the ideal absorption of quercetin glycoside ranges from 3–17%, amounting to a dose of 100 mg. Guo and colleagues [176] suggested that the co-ingestion of fatty acids elevates the bioavailability of quercetin. Quercetins can stabilise and chelate iron and exudes antioxidant properties, which are ascribed to the existence and location of OH^•^ in its chemical constituents. This renders quercetin a free radical scavenger capable of defending against free radical damage. According to Benedetti et al. [177], quercetin modulates the transcription factor AP-1. AP-1 modulates gene expression of various cellular processes such as cell growth and stress. Additionally, quercetin has been reported to activate and induce Sirtuin-1 (SIRT1) activity, associated with mitochondrial formation [178]. Apigenin [179], isorhamnetin [180], and naringin [181] are flavonoids that utilise the blockade of the NF-κB cascade to decrease the generation of inflammatory mediators.

#### 4.2.2. Curcumin

Curcumin, also referred to as diferuloylmethane, is a bright yellow polyphenol, the bioactive substance in the rhizome of Curcuma longa (turmeric) [182]. Some of the multiple health benefits of curcumin include antioxidant, anti-inflammatory, and pain relief and its therapeutic effect on metabolic disorders. The benefits of curcumin as a RONS scavenger are attributed to its chemical structure. Curcumin contains three chemical units in its structure. There are two aromatic ring structures comprising o-methoxy phenolic groups, connected by an α,β-unsaturated β diketone seven-carbon linker [183]. Curcumin is one of the crucial anti-ageing factors. Among the numerous signalling molecules targeted by curcumin is phosphorylase kinase (PhK), which activates and stimulates IFN-γ and NF-κB-dependent signalling cascades and photocarcinogenesis [184]. Anti-carcinogenic properties of curcumin are mainly regulated by the PI3K/Akt/mTOR signalling cascade. In vitro, curcumin has demonstrated neuroprotective properties by averting Aβ protein plaque aggregation. Additionally, curcumin has been observed to hinder NF-κB activation and inflammatory effects. Curcumin regulates SOD, GSH, and CAT activities and prevents ROS-producing enzymes, including xanthine hydrogenase/oxidase and lipoxygenase/cyclooxygenase.

#### 4.2.3. Resveratrol

Resveratrol (3,5,4′-trihydroxystilbene) is a naturally occurring polyphenol compound found in food sources such as seeds, red wine, skins of grapes, peanuts, cranberries, mulberries, and blueberries. As a result of its high metabolism, resveratrol leads to the generation of glucuronides and conjugated sulphates [185]. Some of the functional properties of resveratrol include inducing the upregulation of SOD, GPX, and CAT, averting oxidative DNA damage, and scavenging OH^•^. According to Losso et al. [186], resveratrol regulates ROS generation by activating the AMP-activated protein kinase/SIRT1/proliferator-activated receptor gamma coactivator 1-alpha (AMPK/SIRT1/PGC-1α) signalling cascade to eradicate intracellular dose-dependent downregulation of protein kinase C-beta (PKC-β), TGF-β1, and VEGF. Resveratrol has demonstrated anti-inflammatory, anti-carcinogenic, neuroprotective, anti-thrombotic, and cytoprotective properties, and its consumption remarkably elevates SOD and Nrf2 expressions. According to Gliemann et al. [187], resveratrol suppresses NOX-mediated production of ROS mainly through the downregulation of oxidase expression and activity. Resveratrol reduces the generation of mitochondrial O_2_^•−^, and upregulates the tetrahy-drobiopterin-synthesising enzyme GTP cyclohydrolase I, leading to the prevention of O_2_^•−^ production from disjoined eNOS.

### 4.3. The Antioxidants

#### 4.3.1. Carotenoids

Carotenoids, a broad class of tetraterpenes, are responsible for plant pigmentation. These compounds can be classified into two categories based on their chemical constituents, namely, carotenes and xanthophylls. Carotenes, hydrocarbon-only carotenoids, consist of -carotene, β-carotene, and lycopene. Xanthophylls consist of keto/oxo groups (echinenone and canthaxanthin), oxygen substituents (lutein and zeaxanthin), aldehyde groups (β-citraurin), and epoxide groups (violaxanthin, antheraxanthin, and neoxanthin) [188]. Due to the presence of a polyene constituting an electron-rich conjugated system, carotenoids function as an efficient ROS scavenger. Carotenoids are prominent for their health benefits, such as regulating the immune system, cellular signalling cascades, cellular differentiation, and apoptosis, promoting adhesion molecules and growth factors, and exerting antioxidant properties [189,190]. Due to the highly lipophilic molecules present in carotenoids, they can defend cellular membranes from oxidative stress.

According to Mohammadzadeh Honarvar et al. [191], carotenoids counteract oxidative stress-induced degenerative disorders such as AD and dementia. Carotenoids suppress proinflammatory cytokines, inhibit oxidative stress, and stimulate the production of Aβ peptides, thus impeding the development of diseases. Following consumption, β-carotene is converted to retinol, a readily absorbable form of vitamin A. The beneficial effects of β-carotene include the protective effects against ROS and anti-carcinogenic properties and positively influencing the immune response. On the contrary, Bjelakovic and colleagues [192] indicated that β-carotene elevates CVD and rheumatoid-related mortality rates. Krishnaraj and colleagues [193] reported that β-carotenes bind to AD-related receptors, including the histone deacetylase and P53 kinase receptor, thus exerting antagonistic effects on AD.

#### 4.3.2. Coenzyme 10

Ubiquinone (2,3-dimethoxy-5-methyl-6-polyisoprene parabenzoquinone) or coenzyme 10 is an isoprenoid antioxidant which plays a pivotal role in the ETC. Primary and secondary coenzyme 10 deficiency is correlated with numerous pathological processes such as CVDs, mitochondrial diseases, type II diabetes, cancer, and fibromyalgia [194]. The onset of coenzyme 10 synthesis begins when the isoprenoid building blocks, dimethylallyl pyrophosphate and isopentenyl pyrophosphate, are oligomerised. These building blocks are derived from the key enzyme 3-hydroxy-3-methyl-glutaryl-CoA reductase and the mevalonate cascade [7]. The emerging decaprenyl diphosphate undergoes an amalgamation with a tyrosine derivative, resulting in the formation of the active form of the coenzyme. The active form of coenzyme 10, quinol, regenerates oxidised antioxidants such as vitamins E and C and functions as an ROS scavenger. Additionally, NADPH-dependent systems can reduce the quinone form back to its original form [7].

#### 4.3.3. Vitamins

##### Vitamin C

Vitamin C, also referred to as L-ascorbic acid, is a potent water-soluble vitamin ingested by humans for survival. Vitamin C is a co-factor in pivotal metabolic responses such as collagen synthesis, neuroprotection, iron absorption, and regulating haematopoietic and leukocyte function [195,196]. Furthermore, vitamin C is capable of stabilising and chelating iron. Vitamin C is a free radical scavenger capable of quenching ROS such as O_2_^•−^, OH^•^, H_2_O_2_, HClO, and organic peroxides. Brewer [197] reported that vitamin C impedes oxidation at high concentrations (>1000 mg/kg) by scavenging oxygen. Additionally, vitamin C employs direct or cooperative regeneration of oxidised vitamin E, carotenoids, and GSH to quench ROS.

##### Vitamin E

Vitamin E is a fat-soluble vitamin found naturally in food. Eight forms of vitamin E have been identified, namely α-, β-, γ-, and δ-tocopherol, and α-, β-, γ-, and δ-tocotrienol, based on the hydroxyl and methyl exchange in their phenolic rings. However, α-tocopherol carries the most significant antioxidant properties. Furthermore, α-tocopherol donates a hydrogen atom to numerous ROS, such as O_2_^•−^ and peroxyl radicals. It has been proposed that tocopherols and tocotrienols possess anti-neuroinflammatory and constructive oxidative damage properties. This may suggest that the neuroinflammatory activity of vitamin E includes the stimulation of AD-correlated enzymes such as NOX, COX-2, and 5-lipoxygenase (5-LOX) [198].

## 5. Conclusions

Ageing is a multifactorial phenomenon that negatively influences human health, causing a gradual decline in the body’s normal functionality. With a growing median age in the population, global healthcare systems aim to find answers to alleviating the symptoms, slowing down the progression, and precluding or even treating age-related pathologies. Oxidative stress is characterised by an imbalance in antioxidants and free radicals, contributing to the pathophysiology of numerous human diseases. Oxidative stress facilitates vital pathologies through highly regulated redox-sensitive signalling cascades. While findings from animal and cellular models, coupled with genetic insights, have advanced our knowledge of the molecular mechanisms by which antioxidants attenuate the detrimental effects of ROS, our comprehension is still far from complete. A more detailed evaluation of signalling cascades that lead to chronic degenerative pathologies, suggesting potential intervention targets, is warranted. Over the last few years, there has been growing evidence to suggest that excessive ROS production, such as O_2_^•–^, H_2_O_2_, and OH^•^ radicals, hinder cell growth and induce senescence and programmed cell death. The literature highlighted in the review demonstrates a causative role of oxidative stress in the pathogenesis of CVD, AD, PK, diabetes, and CKD. However, existing antioxidant-based therapies are not target-directed and therefore lack the specificity to promote the reparative response for dysfunctional organelles, cells, and tissue. More compelling clinical translations of antioxidants, such as the efficient concentrations at the target site of oxidative stress, need to be addressed for it to succeed as an effective therapeutic strategy.

## Figures and Tables

**Table 2:** MSC studies in chronic degenerative diseases.

	Cell Line	Studied Model	Tested Parameters	Observations	Ref.
**Alzheimer’s disease**	hMSC	Hippocampal neurons from 18-day-old rat embryos, incubated with hMSC-EVs	Oxidative stress. Neuroprotective action. ROS generation in cultures exposed to AβOs.	hMSC-EVs attenuated AβOs induced robust oxidative stress. Significant elevation of ROS concentrations due to AβOs exposure. Carry active catalase. Block synapse damage.	[138]
	hUMSCs	Male APP/PS1 mice	Spatial probe tests Aβ accumulation assay.	Decrease Aβ generation, oxidative stress and inflammation. Improved memory and cognitive deficits.	[139]
	hUCB-MSCs	Hippocampus of 10-month-old transgenic mouse model.	Cytokine array examination.	Increased release of sICAM-1. Elevated NEP expressions. Decrease in Aβ24 plaques in the hippocampus due to hUCB-MSC migration towards Aβ deposits.	[135]
	hucMSC	AβPP/PS1 transgenic mouse	Behaviour test ELISA for the detection of inflammatory cytokines.	Alleviate neuroinflammation and Aβ deposition. Repair cognitive dysfunctions.	[140]
	BM-MSCs	APP/PS1 mice	Cognitive behaviours. Electrophysiological tests. iNOS mRNA and protein levels.	Improve cognitive behaviour. Decrease synaptic impairment and LTP. Alleviate iNOS expression.	[141]
**Parkinson’s disease**	BM-MSCs	Sprague-Dawley rats, 6 weeks of age	Cell survival, migration, and differentiation of transplanted MSCs. Behavioural observations of PD-model rats. Expression of TH in the SN and the striatum.	BM-MSC transplanted into the lesioned SN, survived, and migrated to other parts of the lesioned brain. Significant improvement in abnormal behaviour following the administration of BM-MSCs. Increase in TH-positive cells in the SN. Increase in the optical density of TH-positive fibres in the striatum.	[142]
	BM-MSCs	Hemiparkinsonian rats	Expression of TH in SN and striatum. Differentiation of MSCs. Analysis of NAA, Cho, and Cr concentrations.	Statistical differences were observed between TH-positive cells in SN and TH-positive terminals in striatum. MSC differentiation into MAP-2-positive neurons. Significant increase in NAA/Cr ratio of 6-OHDA-injected side of the striatum. Significant decrease in Cho/Cr ratio of 6-OHDA-injected side of the striatum.	[143]
	BM-MSCs	Sprague-Dawley rats	In vivo microdialysis Behavioural tests—intensity of rotational behaviour and neurochemical recovery in 6-OHDA lesioned rats.	Group III demonstrated a significant increase in membrane DA transporter and vesicular monoamine transporter-2 compared to group I. Adult MSC reduces behavioural effects induced by 6-OHDA lesions and partially reinstates the vesicular striatal pool and the dopaminergic markers of DA.	[144]
**CVD**	BM-MSCs	Sprague-Dawley rats induced with myocardial infarction	Collagen content. Vascular density.	The combination VEGF/BM-MSCs transplant therapy demonstrated a decrease in collagen content (33%) and a significant elevation in vascular density (80%). BM-MSC transplantation stimulated vascular repair.	[145]
	BM-MSCs	60 patients with ischaemic heart failure	BM-MSCs therapy in patients with severe ischaemic heart failure -a randomised placebo-controlled trial (MSC-HF trial).	Enhanced myocardial function in patients with severe ischaemic heart failure.	[146]
	MSCs	22 patients with non-ischaemic cardiomyopathy with left ventricular ejection fraction	Efficiency and safety of intravenous allogenic MSCs (phase IIa randomised trial).	Immunomodulatory effects. Enhanced functional capacity. MSC therapy was safe.	[147]
**Diabetes**	BM-MSCs	Diabetic Wistar rats	Wound contraction rate. Cellular proliferation. Angiogenesis during wound healing.	Significant reduction in wound sizes, suggesting that BM-MSCs accelerated delayed wound healing BM-MSC transplantation augments cellular proliferation, angiogenesis, and thickens granulation by elevating VEGF expression in delayed wound healing.	[148]
	BM-MSCs	Type II diabetic mice	Blood flow recovery and vasculogenesis. MSC adhesion and migration.	MSCs prestimulated with EGF re-established blood flow recovery and vasculogenesis by promoting neovascularisation by regulating the eNOS, VEGF-A, VEGF/VEGF receptor cascade, and HIF.	[149]
	BM-MSCs	Diabetic rabbit ear ulcer model	Wound closure and angiogenesis.	Allogeneic BM-MSCs improved wound healing by promoting angiogenesis.	[150]
	BM-MSCs	Sprague-Dawley rats	EGF, IGF-1, MMP-2, and pFAK in human keratinocytes.	Improve the keratinocytes by re-established pFAK concentrations and elevating EGF, IGF-1, MMP-2 expressions. Thus reducing the extent of wound healing in DFU on the planar skin of rats.	[151]
**Kidney injury**	BM-MSCs	Mice	Renal function. Cellular proliferation and differentiation.	MSC differentiated into adipocytes. Improved renal function by abrogating tubular damage. Elevated numbers of Ki-67-positive cells, suggesting definite proliferation of MSC, repopulating the injured renal tubule.	[152]
	BMSCs	Adult female mice	Cisplatin-induced injury. Cellular proliferation, migration, and apoptosis.	Decreased severity of cisplatin-induced ARF. Reduced tubular cell apoptosis and augmented tubular cell proliferation. Stimulated proliferation and migration of kidney-derived epithelial cells and elevating cellular survival, thereby restricting renal injury.	[153]
	BM-MSCs	Wistar rats	Cr, FENa, urea, and cytokines. Cellular proliferation.	Diminished Cr, FENa, urea, apoptosis, and necrosis elevations. Elevated cellular proliferation.	[154]

hMSCs, human mesenchymal stem cells; hMSC-EVs, human mesenchymal stem cells-extracellular vesicles; AβOs, amyloid beta oligomers; sICAM-1, soluble intracellular adhesion molecule-1; HUCB-MSCs, human umbilical cord blood stem cells; Aβ, amyloid-β; hUCMSCs, human umbilical cord mesenchymal stem cells; iNOS, inducible nitric oxide synthase; LTP, long-term potential; HIF, hypoxia inducible factor; VEGF, vascular endothelial growth factor; eNOS, endothelial nitric oxide synthase; EGF, epidermal growth factor; IGF-1, insulin-like growth factor; MMP-2, matrix metalloproteinase-2; pFAK, phosphorylated focal adhesion kinase; DFU, diabetic foot ulcers; BM-MSCS, bone marrow mesenchymal stem cells; TH, tyrosine hydroxylase; SN, substantia nigra; NAA, N-acetylaspartate; CHO, choline; CR, creatine; 6-OHDA, 6-hydroxydopamine; DA, dopamine ARF, acute renal failure; BMSC, bone marrow-derived stromal cells.
